# Discriminating Mung Bean Origins Using Pattern Recognition Methods: A Comparative Study of Raman and NIR Spectroscopy

**DOI:** 10.3390/foods14010089

**Published:** 2025-01-01

**Authors:** Mingming Chen, Zhigang Quan, Xinyue Sun, Yanlong Li, Lili Qian, Dongjie Zhang

**Affiliations:** 1 College of Food Science, Heilongjiang Bayi Agricultural University, Daqing 163319, China; chenmingming515@163.com (M.C.); quanzhigang0912@163.com (Z.Q.); 15245806261@163.com (X.S.); a99008191@163.com (Y.L.); 2 Key Laboratory of Agri-Products Processing and Quality Safety of Heilongjiang Province, Daqing 163319, China; 3 National Coarse Cereals Engineering Research Center, Daqing 163319, China

**Keywords:** mung bean, Raman, near-infrared spectroscopy, origin traceability, K-nearest neighbor

## Abstract

The feasibility of the two methodologies was confirmed to compare the results of determining mung bean origins using Raman and Near-Infrared (NIR) spectroscopy. Spectra from mung beans collected in Baicheng City, Jilin Province; Dorbod Mongol Autonomous, Tailai County, Heilongjiang Province; and Sishui County, Shandong Province, China, were analyzed. We established a traceability model using Principal Component Analysis combined with the K-nearest neighbor method to compare the efficacy of these methods in discriminating the origins of the mung beans. The total cumulative variance explained by the first three principal components from the NIR of mung beans from different origins was 99.01%, which is 6.71% higher than that derived from Raman. Additionally, the discrimination rate for mung bean origins based on NIR spectral data reached 98.67%, outperforming the Raman-based approach by 22.67%. These findings indicate that NIR spectroscopy is more effective than Raman spectroscopy is in tracing the provenance of mung beans.

## 1. Introduction

Mung bean (*Vigna radiata*), belonging to the cowpea genus within the legume family and the subfamily *Pterocarpaceae*, is also referred to as green pea or plant pea [1]. At present, the main planting areas of mung beans in China are distributed near the Yellow River and Huai River basins, as well as in the northeast region [2]. Baicheng City, Jilin Province and Dorbod Mongol Autonomous, Tailai County, Heilongjiang Province have been designated as national level geographical indication origins [3]. The unique geographical environment and climate conditions contribute to the distinctive quality of mung beans from various origins and brands. Mung beans taste good; are rich in protein (20–24%), carbohydrate (50–60%), and various vitamins and low in fats (1–2%) [4]; possess health benefits widely recognized by consumers; and have become a highly favored high-quality product in the market [5].

The technology used to trace the origins of agricultural products is essential to an effective quality traceability system. Mass spectrometry [6,7], spectroscopy [8,9], and molecular biology [10] are the primary methods employed to analyze the organic composition, volatile components, isotope contents, and ratios in agricultural products. Combined with chemometric methods, these analyses help establish a distinctive fingerprint spectrum capable of distinguishing and tracing the origins of agricultural products. Consequently, various traceability techniques have significantly enhanced food safety and protected the brand benefits of specialty agricultural products in China.

Raman spectroscopy, often referred to as the Raman effect, was initially theorized by the German physicist Smekall in 1923. He predicted the phenomenon of inelastic light scattering, which the Indian physicist C. V. Raman subsequently demonstrated in 1928. During his research on how light scatters from liquid materials, he identified an inelastic scattering spectrum arising from differences in the rates of molecular polarization [11]. Raman spectroscopy encompasses various techniques, including Dispersive Raman Spectroscopy (DRS), Micro-Confocal Raman Spectroscopy [12], Resonance Raman Spectroscopy [13], and Surface-Enhanced Raman Spectroscopy (SERS) [14]. The advancement of Raman spectroscopy, along with the integration of devices like filters and laser diaphragms, has significantly minimized light scattering. This advancement facilitates the acquisition of high-quality Raman spectra, establishing Raman spectroscopy as a valuable analytical tool in the food industry [15,16]. It boasts several advantages over alternative detection methods, including speed, non-destructiveness, absence of contamination, simplicity, and reproducibility [17,18]. When paired with chemometric methods, it aids in species identification, authenticity screening, and the detection of illegal additions in various foods, including meat [19], cow’s milk and other dairy products [20], aquatic products [21], and honey [22]. For example, Zhu et al. utilized Raman spectroscopy to obtain spectral data from rice samples originating from Heilongjiang, Jilin (Japonica), and Hunan (Indica). Principal Component Analysis (PCA), Hierarchical Cluster Analysis (HCA), and Partial Least Squares Discriminant Analysis (PLS-DA) were integrated to develop a rice-origin discrimination model, achieving an accuracy of 100.00% [23]. Boyacl et al. employed Raman spectroscopy to detect horsemeat adulteration in beef, offering a rapid and accurate method for identifying adulterated meat with minimal analysis time [24]. Additionally, Sha et al. utilized Raman spectroscopy to differentiate rice from three regions—Wuchang, Yanbian, and Panjin-and investigated the impact of grain size on the uniformity of rice flour composition by developing a Support Vector Machine (SVM) model [25]. Raman spectroscopic analysis of phenolics in olive oil, including hydroxytyrosol, as demonstrated by Paiva-Martins et al., highlights the potential of this technique for rapid and nondestructive detection of phenolic compounds [26].

Near-infrared (NIR) spectroscopy is recognized as a fast and efficient technique for spectral analysis in modern science. This technique was initially introduced by Herschel back in 1800. This technique was initially introduced by Herschel back in 1800. NIR light occupies a region of the electromagnetic spectrum with wavelengths between visible light and mid-infrared light. The NIR wavelength range is defined by the American Society for Testing and Materials (ASTM) as 780–2526 nm [27]. In this wavelength range, the interaction of NIR light with C–H and S–H bonds excites hydrogen groups, causing them to transition to higher energy states. As a result, some energy from the NIR light is absorbed by the material being analyzed [28]. This approach is efficient for identifying the composition of different chemical substances and examining their physical properties. Techniques in NIR spectroscopy are noted for their efficiency, speed, non-destructive nature, minimal environmental impact, and cost-effectiveness in detection. NIR spectroscopy has been widely used to trace the origins of various food products, such as citrus fruits [29], kiwifruits [30], honey [31], olive oil [32], wine [33], vinegar [34], and traditional Chinese medicines like Lycium barbarum, forsythia, ephedra, and astragalus [35]. Notable studies include those by Luna et al. utilized NIR spectroscopy to analyze transgenic and non-transgenic soybean oils. By applying multivariate taxonomy with dimensionality and noise reduction, combined with Partial Least Squares Discriminant Analysis (PLS-DA) and Support Vector Machine Discriminant Analysis (SVM-DA), they achieved discrimination accuracies of 100.00% and 90.00% for SVM-DA, and 95.00% and 100.00% for PLS-DA, respectively [36]. Similarly, Ren et al. gathered spectral data on black tea from different origins using NIR spectroscopy combined with factor decomposition methods, achieving a discrimination accuracy of 94.30% [37]. Bevilacqua et al. employed NIR spectroscopy to gather spectral data from 20 certified olive oil samples from Sabina and 37 samples originating from other regions. These data were combined with PLS-DA to develop an origin discrimination model. The results indicated that the model achieved a discrimination accuracy of 100.00% in all cases [38]. Furthermore, Ríos-Reina et al. showed that visible and NIR spectroscopies combined with chemometric analysis effectively distinguish between different white wine varieties [39]. Additionally, there is a lack of comparative studies on the accuracy of these techniques using the same research objects [40]. Few studies have reported the effectiveness of origin traceability techniques that compare Raman and NIR spectroscopy [41].

This study evaluated the impact of Raman and NIR spectroscopy-based methods on the traceability and discrimination of mung beans from different origins. We recorded Raman and NIR spectra from mung bean samples from four origins, performed spectral pre-processing, and used PCA combined with KNN analysis to compare the discriminative models established by both spectroscopic techniques. Our findings provide a theoretical research foundation for future studies on traceability technologies and the development of databases for the origins of agricultural goods.

## 2. Materials and Methods

### 2.1. Sample Collection

**Figure 1:**
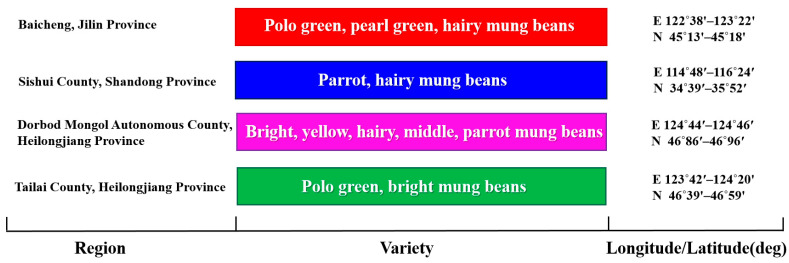
Distribution map of mung bean varieties and sampling points in different regions.

Samples were collected during the first week of the 2021 mung bean harvest (August–September) using a checkerboard sampling method [42]. Test samples (2.0–3.0 kg per site) were taken from regions with recognized geographical indications: Tailai (*n* = 30), Dorbod Mongol Autonomous (*n* = 30), Baicheng (*n* = 30), and Sishui (*n* = 30). Site details and coordinates were recorded with a GPS device (NEO-6M-0-001, Pengrunfa Electronics Co., Shenzhen, China). (Figure 1).

### 2.2. Sample Pretreatment

The mung bean samples were dried in a clean, dust-free environment to remove the husk and debris. The storage conditions were 15–20 °C and 50–60% relative humidity. The samples were ground with a cyclone mill (CT193 CyclotecTM, Guangzhou Yice Instrument Co., Ltd. Guangzhou, China) and passed through a 100-mesh screen to ensure homogeneity.

### 2.3. Spectral Acquisition

Renishaw micro-confocal Raman spectrometer (InVia Reflex; Wilmersin Beijing Science and Technology Co., Ltd., Beijing, China). Collect the Raman spectrum within the range of 100–3200 cm^−1^, with a resolution of 4.5 cm^−1^, 5 s integration time, and laser power 10 mW [43]. 1.0–2.0 g mung bean powder is placed under 100× objective lens, and measured at about 23 °C and 60% humidity in the dark room. Each sample was randomly measured 15 times to reduce experimental error, and the average of three spectra was taken as the final result, producing 600 spectral data points.

Fourier transform near-infrared spectrometer (TENSOR, Bruker Beijing Technology Co., Ltd., Beijing, China) was used with a scanning range of 12,000–4000 cm^−1^, a frequency of 64 times, and a resolution of 8 cm^−1^ [44]. 100 g of mung bean powder was placed in a rotating sampling cup and measured at 25 °C and 45% humidity. The sampling cup was cleaned after each measurement. Each sample was randomly scanned 15 times, and the average of three spectra was taken to generate 600 data points.

### 2.4. Spectral Preprocessing

Various techniques were employed for Raman spectral pre-processing to improve the accuracy and predictive performance of origin discrimination algorithms. The method we developed was based on previous research [45], employing the Moving Average (MA) method [46] and Savitzky-Golay (SG) [47,48] to reduce spectral noise. Adaptive Iterative Re-weighted Penalized Least Squares (AirPLS) [49,50], along with MinMaxScaler (MMS) and the Baseline method, were employed to refine the spectral data. (Table S1).

The NIR spectra underwent several standard pre-processing techniques to ensure data quality and comparability. The method was based on previous research [51] and included Baseline, Spectroscopic, Normalization (Nor) [52], MinMaxScaler (MMS), Centralization (CT), and De-trending (DT) [53]. These methods are crucial for correcting baseline shifts, scaling, and normalizing the spectra to enhance the robustness and reliability of subsequent analyses. (Table S2).

### 2.5. Statistical Analysis

The raw spectral data from Raman and NIR methods were processed using Microsoft Excel 2010 and Origin 2019b. The model’s performance was evaluated using a K-fold cross-validation method (K = 4), where the 600 spectral dataset was divided into four parts. One part was the validation set to test the model’s correct discrimination rate, and the remaining three were used as the training set. Several validation parameters were calculated to assess the performance of the classification models, including Precision, defined as the percentage of true positive predictions among all positive predictions; Recall, defined as the model’s ability to correctly identify samples from each origin; and Accuracy, defined as the overall correctness of the model. The closer the logarithmic value is to 1, the higher the model’s recall or accuracy. The F_1_-score reconciles the tradeoff often encountered between Precision and Recall (2/F_1_ = 1/P + 1/R): high precision usually corresponds to low recall and vice versa.

Python was used to perform PCA for dimensionality reduction of the Raman and NIR spectral data. Subsequently, discriminant models were created by integrating the results with K-nearest neighbor (KNN) analysis. This statistical approach allows for the development of precise and robust origin discrimination models, leveraging the spectral data processed in the previous steps.

## 3. Results

### 3.1. Traceability of the Origin of Mung Beans by Raman Spectroscopy

#### 3.1.1. Acquisition and Pre-Processing of Raw Raman Spectra

**Figure 2:**
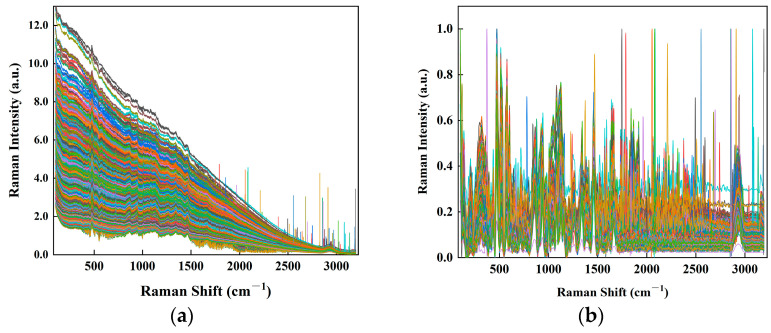
Raw Raman spectra and pre-processed Raman spectra of mung beans from different origins. (**a**) Raman raw spectra of mung beans of different origins; (**b**) Raman spectra of mung beans after sequential processing using Moving Average (MA), Savitzky-Golay (SG), Adaptive Iterative Re-weighted Penalized Least Squares (AirPLS), MinMaxScaler (MMS), and Baseline.

Raman spectra were collected to distinguish mung beans from the four regions of origin. The original Raman spectra (Figure 2a) exhibited substantial baseline correction drift and unsmoothing issues caused by stray light and other factors [54]. Consequently, spectral pre-processing was necessary before constructing the discriminant models. Processed Raman spectrograms, demonstrating the improvement after pre-processing, are depicted in Figure 2b.

#### 3.1.2. PCA Raman Spectra from Mung Beans of Various Origins

**Figure 3:**
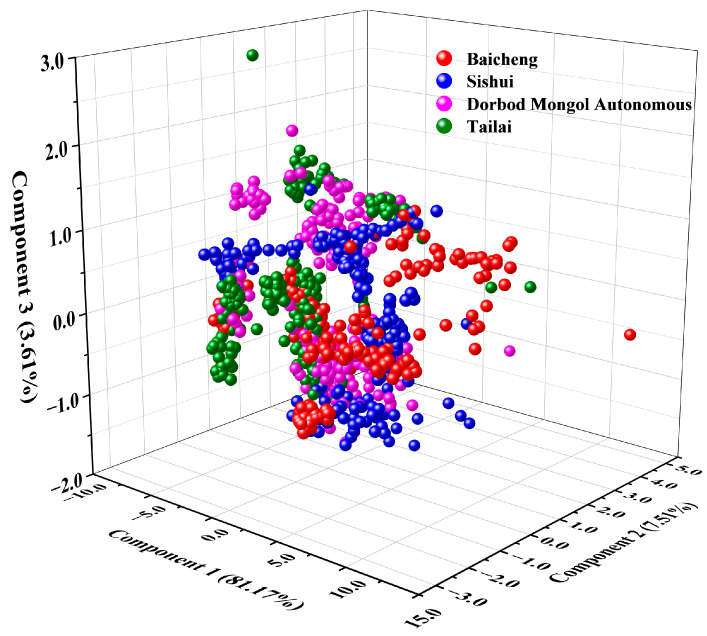
Distribution of mung bean feature vectors for the first three components extracted from PCA features after spectral preprocessing.

Python was employed to analyze the key components of mung bean Raman spectra following a comprehensive series of spectral pre-processing steps (including MA, SG, AirPLS, MMS, and Baseline). Table 1 presents the cumulative variance contribution of each principal component obtained from Raman spectra of mung beans from different origins. As shown in Table 1, the first principal component contributed 81.17% to the cumulative variance, which was the highest among all components. The second and third principal components contributed 7.51% and 3.61% to the variance, respectively, with the total cumulative variance contribution of the top three components reaching 92.30% > 80.00% [55]. Figure 3 shows the feature vector distributions of the first three principal components extracted from the PCA, indicate that the Baicheng samples were spread across different locations, whereas the samples from the other three origins—Tailai, Sishui, and Dorbod Mongol Autonomous—overlapped. This visualization highlights the need for further analysis to effectively categorize and discriminate the origins.

**Figure 4:**
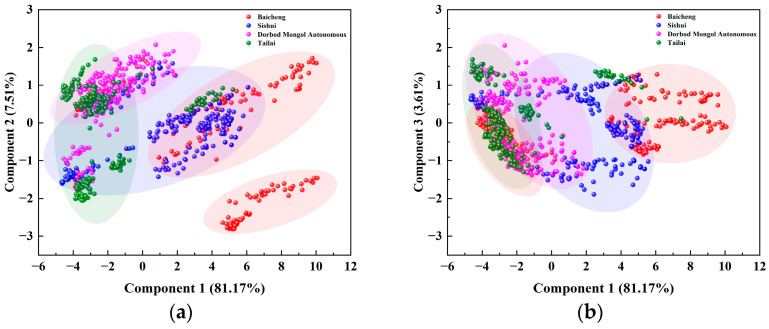
Loadings of PCA on Raman spectra of mung beans from different origins. (**a**) Plot of the first and second principal component loadings of Raman spectra for mung beans from different origins; (**b**) Another plot of the first and second principal component loadings for the same data.

The PCA loading plots of Raman spectra for mung beans from various origins (Figure 4) reveal that the first and second principal components contributed 88.68% to the total cumulative variance, while the first and third components contributed 84.78%. Notably, the scatter plots of the samples originating from the Baicheng and Dorbod Mongol Autonomous regions displayed distinct and autonomous distribution areas, contrasting with the overlapping distributions of Sishui and Tailai. This distinction underscores the effectiveness of PCA in differentiating the Raman spectra of mung beans from these regions, thereby aiding in the precise traceability of their origins. In this study, we used a combination of spectral pre-processing methods including MA, SG, MMS, AirPLS, and Baseline. These methods were instrumental in performing PCA on the Raman spectral data of mung beans from different origins, leading to a total variance contribution of the first three principal components of 92.30%. This approach is consistent with previous studies, such as those by Kuang et al., who utilized comparable pre-processing techniques on vegetable oils and achieved a cumulative variance of 98.01% for the first two principal components [56]. Additionally, Kou et al. achieved a cumulative variance contribution of 85.14% for the first seven principal components in their study of honey using Raman spectroscopy and PCA [57]. These comparisons validate the effectiveness of our selected pre-processing methods.

#### 3.1.3. KNN Analysis of Raman Spectra from Mung Beans of Various Origins

**Figure 5:**
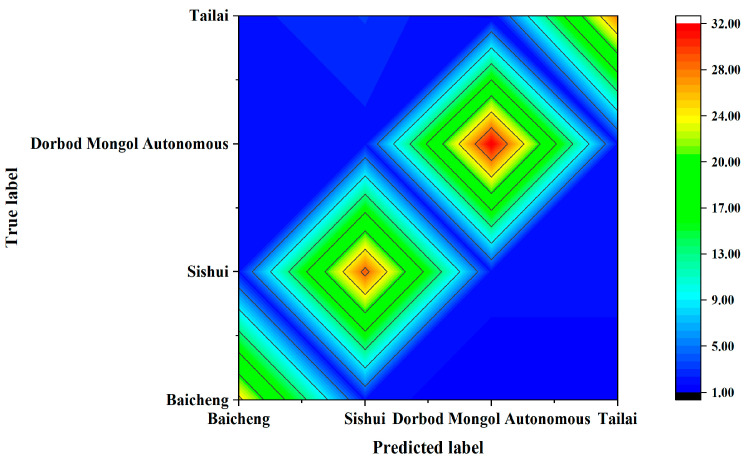
Discriminant matrix of Raman spectra for mung beans from different origins.

The processed spectral data were then utilized to develop a Raman discriminant model for mung beans from different origins using KNN analysis. The results, as shown in Table 2 and depicted in the discrimination matrix (Figure 5), indicate that the PCA–enhanced feature extraction, combined with the KNN approach, successfully discriminated the origins of mung beans with varying accuracy, in decreasing order: Dorbod Mongol Autonomous at 88.89%, Baicheng at 75.76%, Sishui at 74.36%, and Tailai at 66.67% (Dorbod Mongol Autonomous > Baicheng > Sishui > Tailai). The overall rate of origin discrimination stood at 76.00%. These findings highlight the potential of integrating PCA with machine learning methods like KNN to enhance the traceability of agricultural products such as mung beans.

Table 3 shows the accuracy of the mung bean origin classification and identification model based on Raman spectroscopy. The trained model was used to predict the test set data and the performance indicators were calculated to evaluate the identification model based on Raman spectroscopy data.

Table 4 presents the Raman discrimination model assessment indices for mung beans from various origins. It summarizes the results of pre-processing techniques, including PCA, combined with KNN using MA, SG, MMS, AirPLS, and Baseline methods. The model achieved a 75.75% accuracy in discriminating the origins of mung bean samples. The F_1_-score, listed in descending order by origin, were 0.84 (Dorbod Mongol Autonomous), 0.78 (Baicheng), 0.76 (Sishui), and 0.66 (Tailai).

### 3.2. NIR Spectroscopy for Tracing the Origins of Mung Beans

#### 3.2.1. Acquisition and Pre-Processing of Raw NIR Spectra

**Figure 6:**
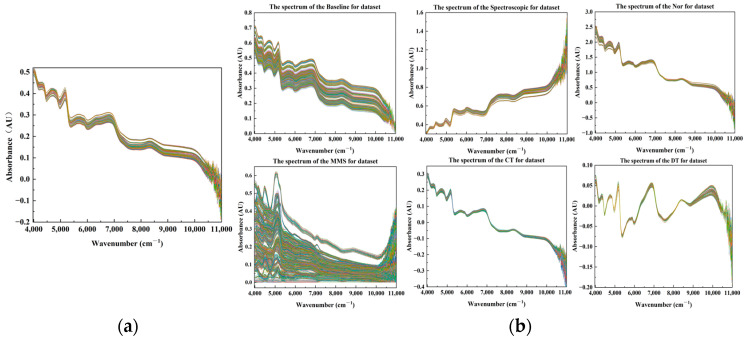
Raw and pre-processed NIR spectra of mung beans from different origins. (**a**) Raw NIR spectra of mung beans from various origins; (**b**) NIR spectra of mung beans processed using six methods: Baseline, Spectroscopic, Normalization (Nor), MinMaxScaler (MMS), Centralization (CT), and De-trending (DT).

NIR spectra were acquired for the four mung bean samples to evaluate two spectroscopic methods for origin tracing using identical research items. The original NIR spectrograms of samples from different origins are displayed in Figure 6a. Owing to the complexity and irregularity of the raw spectra, pre-processing is required during the NIR spectral analysis to mitigate the impact of various elements. Figure 6b shows the pre-processing of the original NIR spectra using six different methods: Baseline, Spectroscopic, Nor, MMS, CT, and DT. These pre-processing techniques enhanced baseline stability and reduced signal fluctuations compared to the original spectra. However, these improvements, except for the Baseline analysis applied to the NIR spectral data of green beans, are not readily visible to the naked eye. Consequently, the processed mung bean NIR spectral data must be utilized to construct subsequent model inputs using the PCA dimensionality reduction method. This approach compares model prediction performance using different input variables and assesses the effectiveness of spectral pre-processing methods for mung bean NIR spectra.

#### 3.2.2. PCA of NIR Spectra for Mung Beans from Different Origins

**Figure 7:**
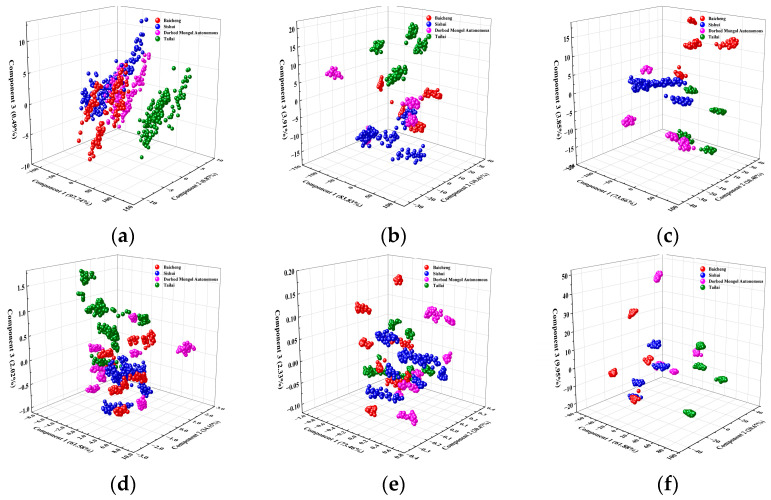
Distribution of mung bean feature vectors for the first three PCA components after various spectral pre-processing methods. (**a**) Baseline. (**b**) Spectroscopic. (**c**) Normalization (Nor). (**d**) MinMaxScaler (MMS). (**e**) Centralization (CT). (**f**) De-trending (DT).

PCA was used for the comparative analysis of NIR spectral pre-treatment on mung bean samples from various origins. The six spectral pre-processing techniques were applied to the NIR spectral data. Table 5 displays the cumulative variance contributions to each primary component of the NIR spectra for various spectral pre-processing techniques. Table 5 indicates that the Baseline method achieves the highest total cumulative contribution among all NIR spectral pre-processing methods. The first principal component accounts for 97.74% of the variance, the second for 0.87%, and the third for 0.49%, resulting in a total cumulative variance of 99.01% for the three components. The three major components’ combined cumulative variance contribution rose by 6.71% compared to the pre-processed Raman spectra. The origin discrimination model in this study used NIR spectral data of mung beans processed with the Baseline method as input variables. Figure 7 displays the mung bean feature vector distributions of the initial three components derived using PCA features following various spectral pre-processing methods. Figure 7 shows that mung bean samples from different origins can be initially identified using the NIR spectral origin feature information extracted using the Baseline spectral pre-processing method. However, the samples from Tailai and Dorbod Mongol Autonomous origins present different spatial distributions, whereas the samples from the other two origins (Baicheng and Sishui) are intersected. After Baseline pre-processing, PCA is applied to downscale the NIR spectral data of mung beans, preserving as much original information as possible to enhance classification and origin discrimination [58].

**Figure 8:**
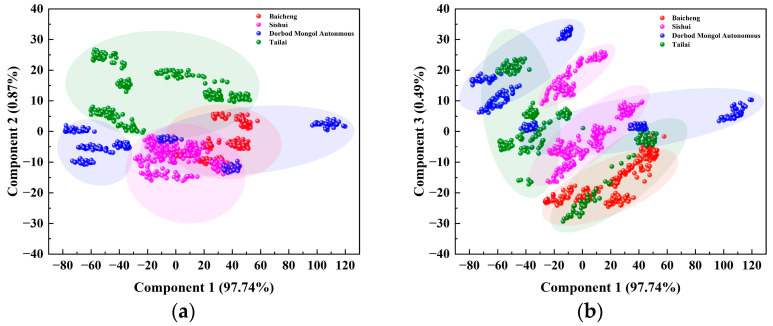
PCA loadings of baseline-processed NIR spectral data for mung beans from different origins. (**a**) Plot of the first and second principal component loadings of NIR spectra for mung beans from different origins; (**b**) Another plot of the first and second principal component loadings for the same data.

Figure 8 presents the PCA loading plots of baseline-corrected NIR spectra for mung beans from different origins, with the first and second principal components accounting for 98.61% of the total variance. The Tailai origin is the only one with an autonomous distribution space; the scatters for the other three origins overlap. The first and third principal components accounted for 98.23% of the total cumulative variance contribution in the loading plots of the first and third principal components. The two roots of Tailai and Dorbod Mongol Autonomous are more discrete in their scattered distribution, whereas the two origins of Baicheng and Sishui are more clearly separated and have autonomous distribution space. In this study, the Baseline preprocessing method was used to perform PCA analysis on the NIR spectral data of mung beans from different origins. The cumulative variance contribution rate of the first three principal components reached 99.01%, which was better than the results of the reference. For example, Yu et al. achieved a cumulative variance contribution of 62.50% for the first two principal components by applying PCA to raw NIR spectra of dried ginger samples pre-processed with SNV+First Derivative and First Derivative methods [59]; Huang et al. performed PCA on raw NIR spectra of imported beef samples from various origins after pre-processing with SNV+First-Order Derivative (FD)+SG [60]. It was found that the first three principal components accounted for 91.32% of the cumulative variance. Thus, KNN analysis can further explore the PCA results of mung bean NIR spectral data from different origins.

#### 3.2.3. KNN Analysis of NIR Spectra for Mung Beans from Various Origins

**Figure 9:**
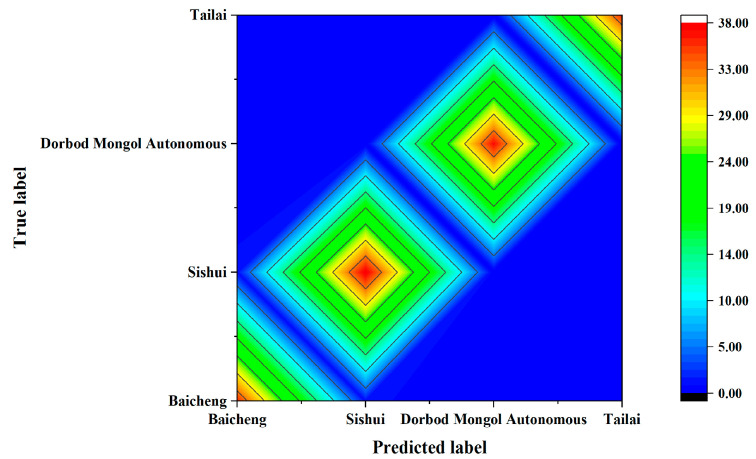
NIR discrimination contour map for mung beans from various origins.

The NIR discrimination model for mung bean samples from different origins was developed by combining KNN with PCA feature extraction following Baseline spectral pre-treatment. Table 6 shows the results of the approach described in Section 3.1.3 for distinct origin classification of mung bean NIR spectra; this information is presented as a contour map in Figure 9. As shown in Table 6 and Figure 9, PCA-derived features were combined with the KNN method to build the NIR discrimination model for tracing the origins of mung beans. The origin determination accuracy was 100.00% for Dorbod Mongol Autonomous and Tailai, 97.30% for Baicheng, and 97.44% for Sishui, with an overall accuracy of 98.67%. The total discrimination of different origins was 22.67% more accurate than that using Raman spectroscopy.

The classification and discrimination accuracy of mung beans from different origins based on NIR is presented in Table 7. The performance index was evaluated by predicting test set outcomes with the trained model to assess the NIR spectral discrimination model. Table 8 presents the evaluation indices for different NIR-based mung bean discrimination models. The table shows that the model achieved an accuracy of 98.50% for mung bean samples from different origins, closely matching the origin discrimination rate, with all F_1_-score exceeding 0.97. The accuracy of this discriminative model showed an improvement of 22.75% and an increase in the F_1_-score by 0.31 compared to the model based on Raman spectral data. This enhancement in model performance can be attributed to integrating data Baseline pre-processing, PCA, and the KNN method in discriminant analysis.

## 4. Discussion

The mung bean samples used in this study were from four regions: Baicheng, Jilin Province; Sishui County, Shandong Province; Dorbod Mongol Autonomous County and Tailai County, Heilongjiang Province. These regions cover the temperate semi-arid zone of Northeast China, the warm temperate monsoon climate zone, and the cold temperate humid zone, representing the growth environment of mung beans under different climate types and soil conditions. Specifically, the average annual temperature in Baicheng is about 4–6 °C, the precipitation is concentrated in summer (400–500 mm). Its soil types are mainly black soil and saline-alkali soil. The average annual temperature in Sishui is about 13 °C, the precipitation is about 800 mm. Its soil types are mainly brown soil and sandy loam. The average annual temperature in Dorbod Mongol Autonomous is 3–4 °C, the annual precipitation is 450–500 mm. The soil conditions are mainly fertile black soil. The average annual temperature in Tailai is about 3 °C, the annual precipitation is about 450 mm. The soil types are mainly black soil and meadow soil. The natural conditions in these areas have a significant impact on the composition and spectral characteristics of mung beans, ensuring the geographical and climatic representativeness of the samples.

Raman and NIR spectroscoies were used to evaluate and compare the traceability identification results of mung beans from different origins. PCA of Raman spectral data showed that the cumulative variance of the first three principal components was 92.30%. PCA of NIR spectral data for mung beans showed a total cumulative variance of 99.01% for the first three principal components, aligning with previous findings. This analysis underscores the superior traceability capabilities of NIR spectroscopy when combined with effective pre-processing and analytical techniques. Additionally, Wu et al. performed quantitative analysis of protein, starch, and moisture in mung beans using Raman and NIR spectroscopies with various spectral pre-processing techniques [61]. Their study on the traceability of mung bean origins compared the effectiveness of two spectroscopic techniques by analyzing the discriminatory models based on Raman and NIR. PCA of Raman spectra from mung beans sourced from different locations indicated that the cumulative variance explained by the first two principal components amounted to 59.80%. In contrast, PCA of the NIR spectra showed a significantly higher cumulative variance contribution of 96.30%. In addition, the accuracy of the origin discrimination model developed in this work using NIR spectroscopy was 98.50%, which is 22.75% higher than that of the model developed using Raman spectroscopy. To evaluate the discriminant model constructed based on NIR spectral data, the accuracy of the model was 98.50%, and the F_1_-score value was above 0.97, which was 0.31 higher than the F_1_-score value of the discriminant model based on Raman spectral data. This substantial difference suggests that NIR spectroscopy is more effective for differentiating the origin of mung beans than Raman spectroscopy is. The lesser efficacy of Raman spectroscopy could be attributed to the Raman effect being inherently weak—approximately 10^−6^ times the intensity of the incident light—making it challenging to identify and analyze weaker Raman scattering signals [62]. Additionally, the low sensitivity of the Raman effect complicates the effective collection of Raman spectra, limiting its practical applications.

Raman spectroscopy, which relies on molecular vibrational modes, yields insights into the vibrations of chemical bonds. This technique is particularly effective for analyzing compounds with intricate molecular architectures like starches, proteins, and aromatic compounds, due to their significant discriminative potential [63]. However, the Raman scattering effect is relatively weak and easily affected by fluorescence effects, which can generate noise in the spectrum, affect signal quality [64], and thus reduce the accuracy of origin discrimination. Therefore, Raman spectroscopy may not be as efficient as NIR spectroscopy for some mung bean samples. NIR spectroscopy operates on the principle of molecular vibrational spectroscopy and involves the selective absorption of NIR by functional groups such as C–H, N–H, and O–H present in the sample. This phenomenon primarily involves the vibrations and rotations of molecules, variations in which can reflect differences between samples from various origins, thus effectively distinguishing their provenance. For example, Ma et al. collected NIR spectra of the lockyang plant from different provinces and used Second-Order Derivative processing combined with chemometrics to determine that lockyang from different origins had strong vibrational absorption peaks at 8408 and 7327 nm, quickly identifying the components of lockyang from different origins [65]. Wu et al. identified characteristic spectral bands utilizing the least squares factor approach, focusing on bands that contained absorption information related to the first- and second-order harmonics, as well as combined frequencies, of the C–H bond. Utilizing the identified characteristic spectral bands, an optimal predictive model was developed [66]. Despite the presence of noise and background disturbances in its spectral data, the application of spectral preprocessing techniques alongside chemometric methods can efficiently remove noise, enhance the detection of characteristic signals, and subsequently increase the accuracy of determining the sample’s origin.

The NIR spectral range reveals the key characteristics of organic components in different types of agricultural products. Samples from different origins have different characteristic information about the organic components, reflecting the influence of various factors such as soil, climate, and environment in different geographical origins. Analyzing the characteristic spectral information can reveal different geographical origins, which can be used for traceability and discrimination research in different geographical origins. Therefore, employing NIR fingerprint analysis technology proves effective in facilitating origin tracing. In our study, the application of NIR spectroscopy for tracing and differentiating the origins of mung beans achieved exemplary outcomes. Our results not only highlight the importance of choosing suitable chemometric techniques but also confirm the viability of using NIR spectroscopy for origin determination in mung beans. At the same time, there is a lack of screening for effective bands related to the origin in our research, which can further explain its effectiveness for origin tracing. For example, Qi et al. gathered raw NIR spectral data from Yunnan Dioscorea, and after preprocessing steps such as normalization and differentiation, they chose second-order derivatives within the range of 1800–400 cm^−1^ for clustering analysis and the Partial Least Squares method. They correctly classified 91.20% of all samples [67]. Jiang et al. used an NIR spectrometer for the rapid detection of peanut acidity. Spectral information was collected within the range of 889.208–1724.71 nm, and the obtained spectral signals were processed by Variable Combination Population Analysis (VCPA) to obtain characteristic bands of peanut acidity. A Support Vector Machine model was developed using the characteristic bands, which demonstrated a correlation coefficient of 0.95 in the results [68]. The favorable correlation coefficient suggests that by choosing characteristic wavelengths associated with origin information, the accuracy, stability, and interpretability of the model can be enhanced. Such improvements aid in more precise identification and differentiation of agricultural products from diverse origins.

In this study, we used PCA combined with KNN method to analyze Raman and NIR spectral data. This combined method showed good performance in spectral analysis with high classification accuracy and reliability. Specifically, PCA can significantly improve the visualization and classification capabilities of high-dimensional data and has been widely used in spectral data processing. In actual classification, the KNN algorithm, owing to its simplicity and efficiency, has been proven to further enhance the classification effect of spectral data when combined with PCA [69]. In comparison, other methods, such as SVM and Random Forest, also have high accuracy in spectral classification. For example, Peng et al. used PCA and SVM to analyze Raman spectra, and the determination coefficient of the PCA–SVM model they established was greater than 0.989 [70]. However, compared with KNN, SVM has higher requirements for parameter optimization and greater computational complexity. Although Random Forest calculations are more robust to data noise, they often require larger sample sizes for training when dealing with high-dimensional data. Therefore, in this study, the method of PCA combined with KNN was proven to be a better choice, especially with a limited sample size.

In addition, the effectiveness of Raman spectral analysis is significantly influenced by the recognition algorithms employed. Advanced techniques such as Deep Learning, Random Forest, SVM, and Cluster Analysis can enhance the discriminative power of Raman spectra. NIR spectroscopy was more effective in this study, a standard NIR system for origin tracing has not yet been established. In addition, origin identification results are affected by factors such as sample quantity, tracing scale, and shelf life, which are rarely reported in origin tracing studies. Thus, although NIR spectroscopy shows promising results, a comprehensive system that incorporates these influencing factors remains crucial for advancing research in origin traceability discrimination. In future studies, we plan to expand the sampling scope to cover other important mung bean-producing areas in China, such as Henan, Anhui, Gansu, and Xinjiang and so on. Inclusion of more sampling regions will help the model capture a wider range of geographical and climatic diversity. Furthermore, increasing the number of samples in each place of origin will ensure adequate statistical representation. The model performance will be further evaluated and optimized through independent dataset validation and external validation (using new samples from other origins), thereby improving the reliability and broad applicability of the results.

## 5. Conclusions

This research utilized Raman and NIR spectroscopic methods to examine mung beans from different origins and sources. After applying various spectral pre-processing methods to these samples, PCA was utilized to examine the NIR spectral data. The analysis demonstrated that the cumulative variance contribution of the first three principal components reached 99.01%. When this PCA–based analysis was combined with the KNN method for further examination of origin traceability, the total correct discrimination rate based on NIR spectroscopy reached 98.67%, which was 22.67% higher than the rate achieved using Raman spectroscopy. This indicates that NIR spectroscopy captured most of the relevant spectral information and accurately reflected the relationship between the principal components and the original data, outperforming the pre-processed Raman spectra by 6.71% in cumulative variance contribution. Moreover, the discriminative models developed using NIR spectral data achieved F_1_-score above 0.97, marking an improvement of 0.31 over those constructed with Raman spectral data. These findings confirm the superior capability of NIR spectroscopy for origin traceability in agricultural products, particularly in differentiating mung bean samples from various origins.

Future research can further explore the potential of Raman and NIR spectroscopies in advancing the traceability of agricultural product origin. Firstly, combined with advanced chemometrics or machine learning algorithms, the classification accuracy of spectral data analysis and the stability of the model can be further improved, especially in dealing with complex data sets and the influence of external environmental factors. Secondly, the complementary characteristics of Raman and NIR spectroscopy are used to combine the advantages of the two technical analysis methods, thereby effectively extracting key spectral features to significantly improve the overall discrimination effect. In addition, exploring the application of portable or on-site spectroscopic instruments can not only promote the development of origin traceability technology, but also provide important technical support for ensuring the quality of agricultural products.

## Figures and Tables

**Table 1:** Cumulative variance contribution of each principal component in the Raman spectra of mung beans from various origins.

Pre-Processing	Component 1 (%)	Component 2 (%)	Component 3 (%)	Total (%)
MA + SG + AirPLS + MinMaxScaler + Baseline	81.17	7.51	3.61	92.30

**Table 2:** Classification results for the origin discrimination of mung bean via Raman spectra.

Origin Classification	Correct Number	Incorrect Number	Accuracy (%)	Total
Baicheng	25	8	75.76	33
Sishui	29	10	74.36	39
Dorbod Mongol Autonomous	32	4	88.89	36
Tailai	28	14	66.67	42

**Table 3:** Accuracy of classification and discrimination models of mung beans from different origins based on Raman.

Classification	Training Set	Validation Set
1	0.7873	0.7575
2	0.7998	0.7687
3	0.7863	0.7560
4	0.7762	0.7477

**Table 4:** Evaluation metrics of the Raman discriminant model for mung beans from different origins.

Origin Classification	Precision (%)	Recall (%)	F_1_-Score	Accuracy (%)
Baicheng	78.00	78.00	0.78	75.75
Sishui	74.00	76.00	0.76	
Dorbod Mongol Autonomous	74.00	86.00	0.84	
Tailai	77.00	65.00	0.66	

**Table 5:** Cumulative variance contributions of each principal component for NIR spectra under different pre-processing methods.

Pre-Processing	Component 1 (%)	Component 2 (%)	Component 3 (%)	Total (%)
Baseline	97.74	0.87	0.49	99.01
Spectroscopic	83.83	10.41	3.91	98.14
Normalization (Nor)	73.66	20.48	3.85	97.78
MinMaxScaler (MMS)	61.58	34.15	2.02	97.75
Centralization (CT)	73.46	20.47	2.33	96.26
De-trending (DT)	61.88	20.67	9.95	92.51

**Table 6:** Classification results for NIR spectral origin discrimination of mung beans from various origins.

Origin Classification	Correct Number	Incorrect Number	Accuracy (%)	Total
Baicheng	36	1	97.30	37
Sishui	38	1	97.44	39
Dorbod Mongol Autonomous	37	0	100.00	37
Tailai	37	0	100.00	37

**Table 7:** Classification and discrimination accuracy of mung beans from various origins based on NIR.

Classification	Training Set	Validation Set
1	0.9878	0.9825
2	0.9896	0.9830
3	0.9860	0.9820
4	0.9878	0.9825

**Table 8:** Evaluation metrics of the NIR discrimination model for mung beans from various origins.

Origin Classification	Precision (%)	Recall (%)	F_1_-Score	Accuracy (%)
Baicheng	97.00	96.00	0.97	98.50
Sishui	97.00	98.00	0.97	
Dorbod Mongol Autonomous	100.00	100.00	1.00	
Tailai	100.00	100.00	1.00	

## Data Availability

The original contributions presented in the study are included in the. article: further inquiries can be directed to the corresponding author.

## Supplementary Materials

The following supporting information can be downloaded at: https://www.mdpi.com/article/10.3390/foods14010089/s1, Table S1: The steps and reasons for Raman spectroscopy pre-processing methods; Table S2: The steps and reasons for NIR spectroscopy pre-processing methods.
