# Supplementary material for: Discriminating Mung Bean Origins Using Pattern Recognition Methods: A Comparative Study of Raman and NIR Spectroscopy
Source: Foods. 2025 Jan 1;14(1):89. doi: 10.3390/foods14010089 (PMC11719895; doi:10.3390/foods14010089)
Supplement: Supplementary file 1 [file foods-14-00089-s001.zip › foods-3374807-supplementary.pdf]

Table S1. The steps and reasons for Raman spectroscopy pre-processing methods

Pre-processing	Steps	Reasons
MA + SG + AirPLS + MMS +Baseline	**(a)** The Raman spectra were smoothed using a sliding window with a window size of n = 5 (which can be adjusted according to the noise level of the data). Reduce random noise by averaging the current data point with the data around it.<br>**(b)** Spectral data were smoothed using a window size of n = 7 and a second-order polynomial fit. SG reduces the impact of noise by fitting a polynomial in a local window and then smoothing the center point.<br>**(c)** The spectrum was baseline corrected using the AirPLS algorithm, which iteratively adjusted weights and minimized the second-order derivative term to adapt to changes in the nonlinear background and ultimately obtain a smooth baseline.<br>**(d)** The preprocessed spectral data is scaled to the range of [0, 1] using the following formula:<br>$$\mathcal{X}' = \frac{\mathcal{X} - \mathcal{X}_{min}}{\mathcal{X}_{max} - \mathcal{X}_{min}}$$<br>Where $\mathcal{X}_{max}$ and $\mathcal{X}_{min}$ are the minimum and maximum values in the spectral data, respectively.<br>**(e)** The overall baseline of the spectrum is corrected using a polynomial fitting method, and the specific steps include: initially fitting a low-order polynomial (e.g., first-order or second-order); Iteratively optimize the fitting results and remove the spectral peak area; Finally, the baseline is subtracted from the original spectrum to obtain the corrected	**(a)** Raman spectra usually contain high-frequency noise, and MA can effectively reduce the impact of noise, improve data quality, and maintain the overall trend of the spectrum.<br>**(b)** It can retain the details and peak position information of the spectral signal while removing noise, and is suitable for characteristic data containing sharp peaks in the Raman spectrum.<br>**(c)** Raman spectroscopy is often interfered by fluorescence background, which causes baseline drift. AirPLS is an adaptive method that can effectively eliminate fluorescence background and thus enhance the visibility of Raman signature peaks.<br>**(d)** The intensity of the Raman spectrum is affected by the instrument settings and the sample concentration. Scaling the data to the same scale range using the MMS method helps to eliminate the amplitude differences between different spectra and enhance the comparability of the models.<br>**(e)** Since the nonlinear drift of the Raman spectral baseline will affect the accurate extraction of characteristic peaks, the Baseline method can effectively remove the low-frequency background and highlight the actual spectral signal.

	spectral data.

Note: **(a)** Moving Average (MA); **(b)** Savitzky-Golay (SG); **(c)** Adaptive Iterative Re-weighted Penalized Least Squares (AirPLS); **(d)** MinMaxScaler (MMS); **(e)** Baseline.

Table S2. The steps and reasons for NIR spectroscopy pre-processing methods

Pre-processing	Steps	Reasons
Baseline	**(a)** The baseline is estimated by a polynomial fitting method (e.g., a second-order polynomial) and subtracted from the original spectrum to remove low-frequency drifts of the spectrum.	**(a)** Since the baseline drift of spectral data may come from instrument noise and environmental influences, Baseline can effectively remove the drift and highlight the true signal characteristics of near-infrared spectroscopy.
Spectroscopic	**(b)** The spectral data were processed using first- and second-order derivative transformations (e.g., the Savitzky-Golay), with the window size n=11 and the polynomial order *p*=2.	**(b)** Derivative transformations can help enhance subtle changes in spectra, remove background interference, and highlight the characteristics of absorption peaks. In addition, the second-order derivative can further reduce the baseline effect and improve the resolution of characteristic peaks.
Nor	**(c)** The spectral data were normalized. The formula is as follows: $$\mathcal{X}' = \frac{\mathcal{X} - \mu}{\sigma}$$ Where μ is the mean and σ is the standard deviation.	**(c)** The amplitude differences of spectral data can be eliminated, so that the data of different samples can be compared within the same scale range, thereby improving the model performance.
MMS	**(d)** Scale the spectral data to the range [0, 1]. The formula is as follows: $$\mathcal{X}' = \frac{\mathcal{X} - \mathcal{X}_{min}}{\mathcal{X}_{max} - \mathcal{X}_{min}}$$ Where $\mathcal{X}_{max}$ and $\mathcal{X}_{min}$ are the minimum and maximum values in the spectral data, respectively.	**(d)** It helps to eliminate amplitude differences between samples, especially for algorithms that require model inputs to be in the same range, such as Support Vector Machines (SVM).
CT	**(e)** Subtract the mean of each spectrum to center the data distribution around zero. The formula is as follows: $$\mathcal{X}' = \mathcal{X} - \mu$$ Where μ is the mean of the spectral data.	**(e)** It helps to remove the offset of the data, enhance the contrast of the spectral data, and make it easier for the algorithm to capture characteristic changes in the spectrum.
DT	**(f)** The spectral data are corrected for low-frequency trends using a detrending algorithm by fitting a	**(f)** Effectively remove the overall trend changes caused by instrument drift or environmental influences,

	low-order polynomial (e.g., a first-order straight line) and subtracting the trend from the original data.	ensuring that the local characteristic information of the spectrum will not be masked by the global trend.

Note: **(a)** Baseline; **(b)** Spectroscopic; **(c)** Normalization (Nor); **(d)** MinMaxScaler (MMS); **(e)** Centralization (CT); **(f)** De-trending (DT).
